# Supplementary material for: Protective MVA-ST Vaccination Robustly Activates T Cells and Antibodies in an Aged-Hamster Model for COVID-19
Source: Vaccines (Basel). 2024 Jan 3;12(1):52. doi: 10.3390/vaccines12010052 (PMC10819389; doi:10.3390/vaccines12010052)
Supplement: Supplementary file 1 [file vaccines-12-00052-s001.zip › vaccines-2763404-supplementary.pdf]

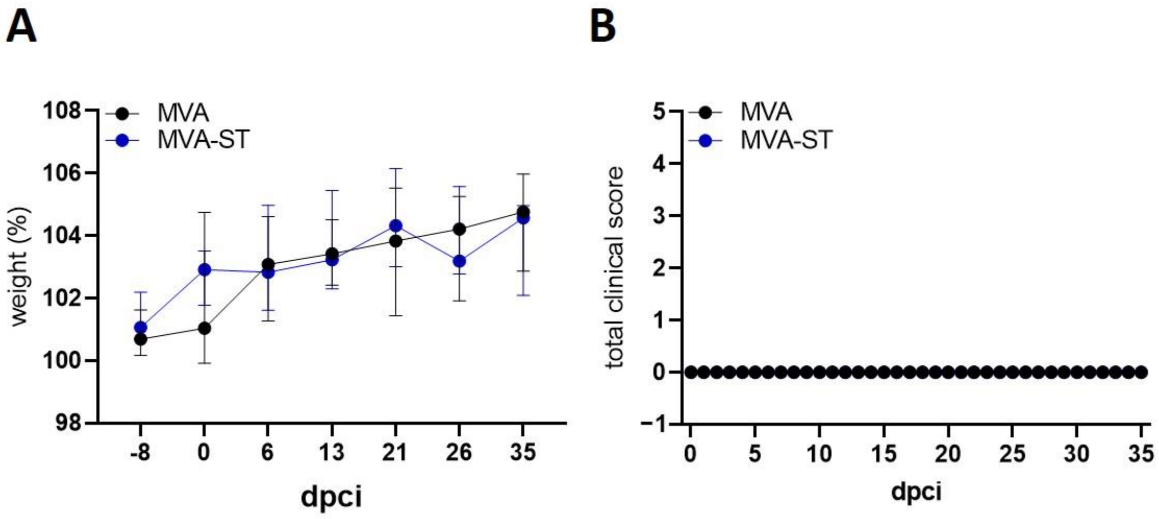

Figure S1. MVA ST-immunization and monitoring for side effects in aged hamsters. Groups of hamsters were vaccinated twice, via the intramuscular route, in a 21-day interval with 10^8 PFU of either non-recombinant MVA or MVA expressing the stabilized version of the SARS-CoV-2 S protein (MVA-ST). All Hamsters were monitored daily for body weight changes (A) and clinical symptoms (B) after each vaccination.

SARS-CoV-2 N protein of USA-WA1/2020

Peptides 17- to 13-mer, with 10 peptides overlap

MSDNGPQNQR NAPRITFGGP SDSTGSNQNG ERSGARSKQR RPQGLPNNTA SWFTALTQHG KEDLKFPRGQ GVPINTNSSP DDQIGYYRRA TRRIRGGDGK MKDLSPRWYF YYLGTGPEAG LPYGANKDGI IWVATEGALN TPKDHIGTRN PANNAAIVLQ LPQGTTLPKG FYAEGSRGGS QASSRSSSRS RNSSRNSTPG SSRGTSPARM AGNGGDAALA LLLLDRLNQL ESKMSGKGQQ QQGQTVTKKS AAEASKKPRQ KRTATKAYNV TQAFGRRGPE QTQGNFGDQE LIRQGTDYKH WPQIAQFAPS ASAFFGMSRI GMEVTPSGTW LTYTGAIKLD DKDPNFKDQV ILLNKHIDAY KTFPPTEPKK DKKKKADETQ ALPQRQKKQQ TVTLLPAADL DDFSKQLQQS MSSADSTQA

SARS-CoV-2 S protein of USA-WA1/2020

Peptides 17- to 13-mer, with 10 peptides overlap

MFVFLVLLPL VSSQCVNLTT RTQLPPAYTN SFTRGVYYPD KVFRSSVLHS TQDLFLPFFS NVTWFHAIHV SGTNGTKRFD NPVLPFNDGV YFASTEKSNI IRGWIFGTTL DSKTQSLLIV NNATNVVIKV CEFQFCNDPF LGVYYHKNNK SWMESEFRVY SSANNCTFEY VSQPFLMDLE GKQGNFKNLR EFVFKNIDGY FKIYSKHTPI NLVRDLPQGF SALEPLVDLP IGINITRFQT LLALHRSYLT PGDSSSGWTA GAAAYYVGYL QPRTFLLKYN ENG-TITDAVD CALDPLSETK CTLKSFTVEK GIYQTSNFRV QPTESIVRFP NITNLCPFGE VFNATRFASV YAWNRKRISN CVADYSVLYN SASFSTFKCY GVSPTKLNDL CFTNVYADSF VIRGDEVRQI APGQTGKIAD YNYKLPDDFT GCVIAWNSNN LDSKVGGNYN YLYRLFRKSN LKPFERDIST EIYQAGSTPC NGVEGFNCYF PLQSYGFQPT NGVGYQPYRV VVLSFELLHA PATVCGPKKS TNLVKNKCVN FNFNGLTGTG VLTESNKKFL PFQQFGRDIA DTT-DAVRDPQ TLEILDITPC SFGGVSVITP GTNTSNQVAV LYQDVNCTEV PVAIHADQLT PTWRVYSTGS NVFQTRA GCL IGAEHVNNSY ECDIPIGAGI CASYQTQTNS PRRARSVASQ SIIAYTMSLG AENSVAYSNN SIAIPTNFTI SVTTEILPVS MTKTSVDCTM YICGDSTECS NLLLQYGSFC TQLNRALTGI AVEQDKNTQE VFAQVKQIYK TPPIK-DFGGF NFSQILPDPS KPSKRSFIED LLFNKVTLAD AGFIKQYGDC LGDIAARDLI CAQKFNGLTV LPPLLTDEMI AQYTSALLAG TITSGWTFGA GAALQIPFAM QMAYRFNGIG VTQNVLYENQ KLIANQFNSA IGKIQDSLSS TASALGKLQD VVNQNAQALN TLVKQLSSNF GAISSVLNDI LSRLDKVEAE VQIDRLITGR LQSLQTYVTQ QLI-RAAEIRA SANLAATKMS ECVLGQSKRV DFCGKGYHLM SFPQSAPHGV VFLHVTYVPA QEKNFTTAPA ICHDGKAHFP REGVFVSNGT HWFVTQRNFY EPQIITTDNT FVSGNCDVVI GIVNNTVYDP LQPELDSFKE ELDKYFKNHT SPDVDLGDIS GINASVVNIQ KEIDRLNEVA KNLNESLIDL QELGKYEQYI KWPWYIWLGF IAGLI-AIVMV TIMLCCMTSC CSCLKGCCSC GSCCKFDEDD SEPVLKGVKL HYT

Figure S2. Protein sequence of SARS-CoV-2 nucleoprotein (N) and SARS-CoV-2 spike protein (S) used for splenocyte stimulation. The SARS-CoV-2 nucleoprotein comprises of 419 amino acids (aa). For hamster splenocyte stimulation, one peptide pool, consisting of 59 overlapping peptides, were derived from the SARS-CoV-2 N protein sequence. Each single peptide consists of 17 or 13 amino acids (17- or 13-mers) overlapping in 10 amino acids with the following peptide. The SARS-CoV-2 spike glycoprotein comprises 1273 aa. For hamster splenocyte stimulation, two peptide pools (S1, underlined in yellow, and S2, underlined in blue, amino acids underlined in green present in both pools), consisting of 91 and 90 overlapping peptides, were derived from the SARS-CoV-2 S protein sequence. Each single peptide consists of 17 or 13 amino acids (17- or 13-mers) overlapping in 10 amino acids with the following peptide.
